# The contribution of CXCL12-expressing radial glia cells to neuro-vascular patterning during human cerebral cortex development

**DOI:** 10.3389/fnins.2014.00324

**Published:** 2014-10-15

**Authors:** Mariella Errede, Francesco Girolamo, Marco Rizzi, Mirella Bertossi, Luisa Roncali, Daniela Virgintino

**Affiliations:** Department of Basic Medical Sciences, Neurosciences and Sensory Organs, University of Bari School of MedicineBari, Italy

**Keywords:** neuroangiogenesis, blood-brain barrier, tight junctions, transporters, radial glia, chemokine CXCL12, connexin 43, human fetus

## Abstract

This study was conducted on human developing brain by laser confocal and transmission electron microscopy (TEM) to make a detailed analysis of important features of blood-brain barrier (BBB) microvessels and possible control mechanisms of vessel growth and differentiation during cerebral cortex vascularization. The BBB status of cortex microvessels was examined at a defined stage of cortex development, at the end of neuroblast waves of migration, and before cortex lamination, with BBB-endothelial cell markers, namely tight junction (TJ) proteins (occludin and claudin-5) and influx and efflux transporters (Glut-1 and P-glycoprotein), the latter supporting evidence for functional effectiveness of the fetal BBB. According to the well-known roles of astroglia cells on microvessel growth and differentiation, the early composition of astroglia/endothelial cell relationships was analyzed by detecting the appropriate astroglia, endothelial, and pericyte markers. GFAP, chemokine CXCL12, and connexin 43 (Cx43) were utilized as markers of radial glia cells, CD105 (endoglin) as a marker of angiogenically activated endothelial cells (ECs), and proteoglycan NG2 as a marker of immature pericytes. Immunolabeling for CXCL12 showed the highest level of the ligand in radial glial (RG) fibers in contact with the growing cortex microvessels. These specialized contacts, recognizable on both perforating radial vessels and growing collaterals, appeared as CXCL12-reactive *en passant, symmetrical* and *asymmetrical*, vessel-specific RG fiber swellings. At the highest confocal resolution, these RG varicosities showed a CXCL12-reactive dot-like content whose microvesicular nature was confirmed by ultrastructural observations. A further analysis of RG varicosities reveals colocalization of CXCL12 with Cx43, which is possibly implicated in vessel-specific chemokine signaling.

## Introduction

Radial glial (RG) cells were first described in the developing CNS by Magini and Ramón y Cajal in the late 19th century (Bentivoglio and Mazzarello, 1999). Their function as scaffolding for neuroblast migration during cortical histogenesis was postulated and demonstrated by Rakic in the early 1970s (Rakic, 1971, 1972) and then, at the beginning of the new millennium, their role as neuronal and glial precursors in the developing CNS was definitively demonstrated (Malatesta et al., 2000; Malatesta and Götz, 2013). The evidence that RG cells fulfill many functions has attracted new attention to these cells and new discoveries about their diverse functions in the developing brain are rapidly accumulating.

RG cells differ taxonomically in primates and other mammalian species due to differences in their involvement in neurogenesis, and the timing and region of their appearance, as regards the expression of typical RG immunomarkers (Howard et al., 2008; Xu et al., 2014). For example, in humans, the intermediate filament proteins, glial fibrillary acidic protein (GFAP), and vimentin, are expressed concomitantly in RG from the start of neurogenesis (Choi and Lapham, 1978; Virgintino et al., 1998; Howard et al., 2006). In contrast, in rodents, RG cells undergo a more protracted maturation and become GFAP-positive only at late fetal stages, after neurogenesis is complete, and vimentin is no longer detectable (Rickmann and Wolff, 1985). Moreover, in human telencephalon, due to the larger size of the cerebral cortex compared with the rodent cortex, a “mature” GFAP-expressing RG precociously develops long shafts that may reach a length of 3000–7000 μm and show early, special vascular relations (Rakic, 1972; Virgintino et al., 1998).

Pioneering studies have demonstrated that angiogenically formed microvessels invade the developing brain by perforating the basement membrane and penetrating through the external glial limiting membrane to supply the developing nervous tissue, under the control of a concentration gradient of soluble growth factors (Risau et al., 1988; Risau and Wolburg, 1990; Engelhardt and Risau, 1995). In humans the growing microvessels arrange themselves according to geometrically precise paths that parallel the RG arrangement, taking on a common radial pattern and forming mutual, multiple contacts (Bär, 1980; Marin-Padilla, 1985; Virgintino et al., 1998; Rakic, 2007; Marín-Padilla, 2012). In recent years it has become increasingly evident that the role of RG cells during brain vascularization may be more specific than was previously thought. In fact, besides a simple activity providing tracks for growing vessels, RG cells have been suggested to be the source of factors such as vascular endothelial growth factor (VEGF) and Wnt, that intertwine with each other and other angiogenic pathways to facilitate the guidance of penetrating microvessels, stimulate proliferation of endothelial cells (ECs) and sustain vessel stabilization (Liebner and Plate, 2010; Bussmann et al., 2011; Quaegebeur et al., 2011).

During human cerebral cortex vascularization, subsets of RG cells and perivascular astrocytes have been demonstrated, showing high levels of chemokine CXCL12 expression (Virgintino et al., 2013). This chemokine, also known as stromal-derived factor-1 (SDF-1), plays multiple roles in CNS development, its expression in the brain being involved in neural progenitor differentiation, and neuronal cell migration (Tiveron et al., 2006; Peng et al., 2007; Stumm and Höllt, 2007; Li et al., 2008; Tiveron and Cremer, 2008). Moreover, cells of neural crest origin, located in the developing, innermost layer of the meninges, have been demonstrated to be involved in neurogenesis, secreting molecular cues including chemokine CXCL12, that seem to regulate cortical interneuron settlement, and Cajal–Retzius cell tangential migration (Stumm et al., 2003; Borrell and Marín, 2006; Paredes et al., 2006; Li et al., 2008; Siegenthaler et al., 2009).

**Figure 1:**
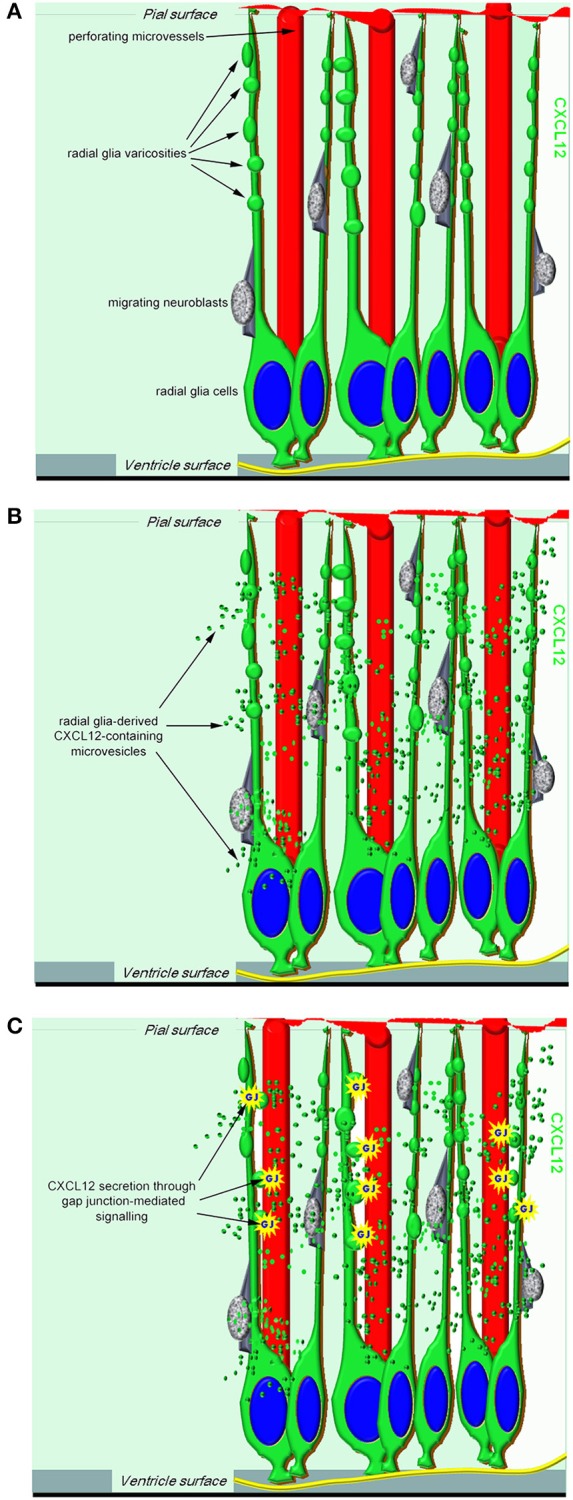
**A proposed model of RG-derived CXCL12 regulation of cerebral cortex development and vascularization. (A)** In the working model, CXCL12 radial glia cells have a central role not only in neurogenesis and neuroblast migration but also in neuroangiogenesis, being ideally positioned to coordinate neuro-vascular cerebral cortex patterning. **(B)** Radial glia-derived CXCL12-containing microvesicles may convey their signal to the ECs of growing microvessels. **(C)** One potential mechanism by which CXCL12 secretion can be modulated in radial glia cells is through gap junction-mediated signaling.

In addition, CXCL12 and its receptor CXCR4 (C-X-C chemokine receptor) belong to a restricted group of pro-angiogenic molecules that, albeit within a limited developmental window, display a neuro-specific angiogenic activity (Bussmann et al., 2011; Quaegebeur et al., 2011). Overall, these data are consistent with previous observations of human developing cerebral cortex, which have described CXCR4/CXCR7-reactive neuroblasts and subsets of CXCL12 RG cells and astrocytes in close association with CXCR4/CXCR7-reactive microvascular ECs and pericytes (Virgintino et al., 2013; see also Table 1). Accordingly, a model of synchronized neuro-vascular patterning centered on CXCL12 RG cells has been proposed (Figure 1) and appears to be supported by the present results that have identified, by parallel laser confocal and electron microscopy analyses, RG-microvessel subcellular units shaped by RG varicosities containing CXCL12 microvesicles (MVs) and connexin 43 (Cx43) as a regulatory molecule.

**Table 1:** **Synopsis of CXCL12 and CXCR4/CXCR7 immunoreactivity in developing brain**.

**Cell type**	**CXCL12**	**CXCR4**	**CXCR7**
Radial glia cells	cytoplasm^high^	-	-
Perivascular astrocytes	cytoplasm^high^	-	-
Neuroblasts	-	nucleus^high^	nucleus^high^
Endothelial cells	cytoplasm^low^	-	-
Pericytes	-	cytoplasm^high^	cytoplasm^high^

Modified from Virgintino et al. (2013).

## Materials and methods

### Histology

Autopsy specimens of telencephalon were collected from 4 human fetuses spontaneously aborted due to preterm rupture of the placental membranes (22 weeks of gestation). The sampling and handling of human specimens conformed to the ethical rules of the Department of Pathology, Medical School, University of Bari, Italy, and approval was gained from the local Ethics Committee of the National Health System in compliance with the principles stated in the Declaration of Helsinki. The fetal age was estimated on the basis of the crown-rump length and/or pregnancy records (counting from the last menstrual period). At autopsy, the selected fetuses did not reveal macroscopic structural abnormalities and/or malformations of the central nervous system. The dorso-lateral wall of the telencephalic vesicles (future neocortex) was dissected and fixed for 2–3 h at 4°C by immersion in 2% paraformaldehyde plus 0.2% glutaraldehyde solution. Specimens were then washed in phosphate buffered saline (PBS, pH 7.6) and serially sectioned by a vibrating microtome. 20 μm sections were collected at regular intervals and processed for conventional histological analysis with toluidine blue staining to ascertain the absence of microscopic malformations; all the other sections were stored in PBS plus 0.02% paraformaldehyde for fluorescence immunolabeling.

### Immunofluorescence and laser confocal microscopy

Multiple immunostainings were carried out with the following polyclonal antisera (pAbs) and monoclonal antibodies (mAbs), all diluted in blocking buffer (1% bovine serum albumin and 2% fetal calf serum in PBS): rabbit pAb anti-Glut 1, mouse mAb anti-P-gp, mouse mAb anti-Claudin-5, rabbit pAb anti-Occludin, mouse mAb anti-CXCL12/SDF-1, rabbit pAb anti-CD105, rabbit pAb anti-NG2 (generous gift from W. B. Stallcup, The Burnham Institute for Medical Research, La Jolla, CA, USA), mouse mAb anti-GFAP, rat mAb anti-GFAP, rabbit pAb anti-collagen type IV (Coll IV), rabbit pAb anti-Cx43 (Table 2). After adhesion on polylysine slides (Menzel-Glaser, GmbH, Braunschweig, Germany) by drying for 10 min at room temperature, the sections were submitted to the following protocols: rehydration with PBS for 5 min at room temperature or microwave pre-treatment in 0.01M citrate buffer (pH 6.0) for 15 min at 750W when required (Table 2); incubation with 0.5% Triton X-100 in PBS for 30 min at room temperature and with blocking buffer 30 min at room temperature, with single or combined primary antibodies overnight at 4°C, then with appropriate fluorophore-conjugated secondary antibodies (Table 2). Finally, the sections were washed for 10 min × 3 in PBS between each step, counterstained with either TO-PRO-3™ (1:10K dilution; Invitrogen Corporation, Carlsbad, CA, USA) or Sytox® Green (1:5K dilution; Invitrogen), mounted in Vectashield (Vector Laboratories, Inc. Burlingame, CA, USA), and finally sealed with nail varnish. Negative controls were prepared by using primary antibodies to stain inappropriate tissues, omitting the primary antibodies, pre-adsorbing the primary antibodies with an excess of antigen when available, or mismatching the secondary antibodies on appropriate preparations. When not ascertained in previous experiments, the specificity of primary antibodies was tested on tissue sections known to contain the antigen, as positive controls, and applying the same immunostaining protocols described above. Human adult brain sections were used to test the BBB-specific markers (Glut 1, P-gp, Claudin-5, Occludin), Cx43 antibody was tested on human and mouse brain sections and on mouse heart sections, while sections from human tonsils were useful for assessing CXCL12 and CD105, immunoreactivity (control sections were kindly provided by E. Maiorano, Department of Emergency and Organ Transplantation, Pathological Anatomy Unit, Faculty of Medicine, University of Bari, Bari, Italy). The sections were examined under the Leica TCS SP5 confocal laser-scanning microscope (Leica Microsystems, Mannheim, Germany) using a sequential scan procedure. Confocal images were taken at 250 nm intervals through the z-axis of the sections. Confocal images were taken with 40X and 63X oil lenses. Z-stacks of serial optical planes (projection images) and single optical planes were analyzed by Leica confocal software (Multicolor Package; Leica Microsystems). The size of RG varicosities and MVs was evaluated with LAS-AF SP5 software (Leica Microsystems) on 63X magnification fields zoomed 3 times. MVs diameter (nm) was measured on single optical planes from CXCL12-labeled sections (*n* = 18) for a total of 50 fields. The results were expressed as a “range” of size from a minimum to a maximum value.

**Table 2:** **List of primary and secondary antibodies combined in multiple immunolabelings**.

**Primary antibodies**	**Host IgG**	**Dilution**	**Antigen retrieval**	**Producer**	**Code number**
anti-Glut 1	rabbit IgG	1:100	–	Millipore^a^	07-1401
anti-P-gp	mouse IgG_2a_	1:10	–	Signet^b^	8720-01 C494
anti-Claudin-5	mouse IgG_1_	1:20	MW	Zymed^c^	18-7364 4C3C2
anti-Occludin	rabbit IgG	1:50	–	Zymed^c^	71-1500
anti-CXCL12/SDF-1	mouse IgG_1_	1:10	MW	R&D Systems^d^	MAB350
anti-CD105	rabbit IgG	prediluted	MW	Abcam^e^	ab27422
anti-NG2	rabbit IgG	1:50	–	W.B. Stallcup	–
anti-GFAP	mouse IgG_1_	1:50	–	Novocastra^f^	NCL-GFAP-GA5
anti-GFAP	rat IgG_2a_	1:50	–	Invitrogen^g^	13-0300
anti-Coll IV	rabbit IgG	1:50	–	Acris^h^	R1041
anti-Cx43	rabbit IgG	1:50	MW	Millipore^a^	AB1728
**Secondary antibodies**		**Dilution**		**Producer**	**Code number**
1 goat anti-rabbit Alexa 555		1:300		Invitrogen^g^	A21429
2 goat anti-mouse Alexa 555		1:300		Invitrogen^g^	A21425
3 goat anti-mouse Alexa 488		1:300		Invitrogen^g^	A11001
4 goat anti-rabbit Alexa 488		1:300		Invitrogen^g^	A11070
5 goat anti-rat Alexa 555		1:300		Invitrogen^g^	A21434
6 goat anti-mouse Alexa 633		1:300		Invitrogen^g^	A21126

Glut 1/GFAP (revealed by 1, 3); Occludin/P-gp (1, 3); Occludin/Claudin-5 (1, 3); CD105/CXCL12 (4, 2); CXCL12/GFAP/Coll IV (6, 5, 4); CXCL12/Cx43 (3, 1).

a Millipore-Chemicon; Billerica, MA, USA.

b Signet Laboratories, Dedham, MA, USA.

c Zymed Laboratories, Invitrogen Corporation, Carlsbad, CA, USA.

d R&D Systems, Minneapolis, MN, USA.

e Abcam, Cambridge, UK.

f Vision Biosystem Novocastra, Newcastle upon Tyne, UK.

g Invitrogen, Eugene, OR, USA.

h Acris Antibodies GmbH; Herford, Germany. MW, high-temperature microwave pre-treatment of tissue sections.

### Transmission electron microscopy

Small samples collected from the previously described telencephalon specimens were submitted to electron microscopy procedures. Briefly, samples were fixed in 0.1 M phosphate-buffered 3% glutaraldehyde, post-fixed in phosphate-buffered 1% OsO4, dehydrated in serial alcohols, and embedded in Epon 812. Ultrathin sections were cut with an LKB V ultramicrotome (LKB Bromma, Sollentuna, Sweden), stained with uranyl acetate and lead citrate, and observed under a CM 10 Philips electron microscope (Philips, Eindhoven, The Netherlands).

## Results

### Cerebral cortex microvessels show a BBB-specific phenotype

**Figure 2:**
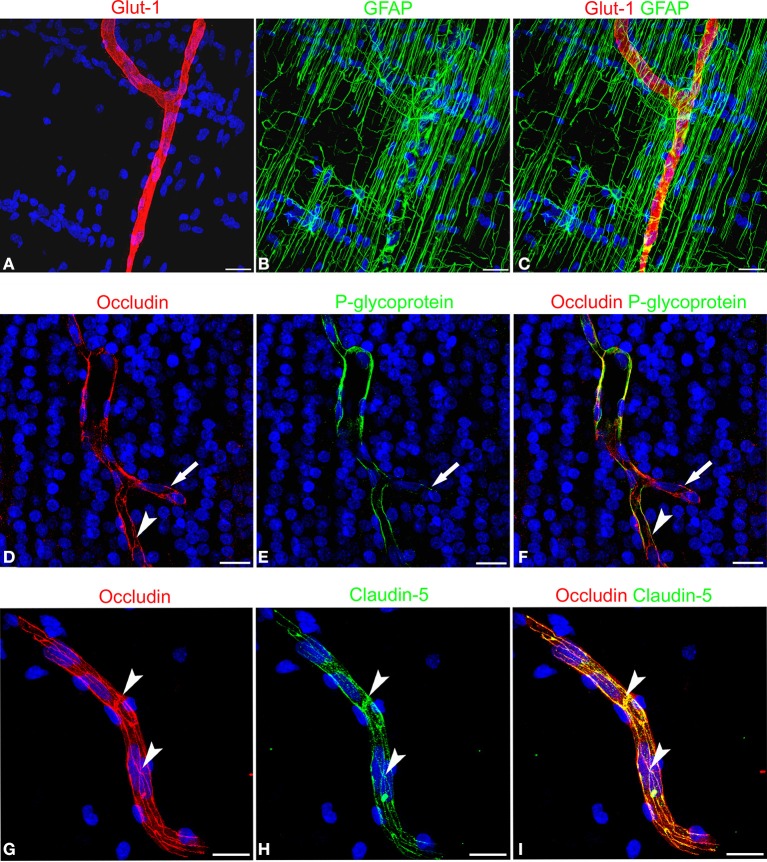
**Confocal images of human cerebral cortex at midgestation double labeled with Glut-1/GFAP, occludin/P-glycoprotein, and occludin/claudin-5**. **(A–C)** A radial microvessel surrounded by GFAP-reactive RG fibers is revealed by the Glut-1 reactive endothelial cells. **(D–F)** On a stem vessel and its collateral (*arrow*) endothelial P-glycoprotein colocalize with occludin reactivity; note in **(E)** the lower levels of P-glycoprotein expression on the newly formed vessel branch (*arrow*) and in **(D)** and **(F)** the junctional linear pattern of occludin (*arrowhead*). **(G–I)** Occludin appears arranged according to a typical junctional pattern and colocalizes at points with claudin-5 (*arrowheads*). Bars: **A–C** and **D–F** 25 μm; **G–I** 30 μm.

The expression of blood-brain barrier (BBB)-specific markers was analyzed in microvessels that, at the end of neuronal migration and beginning of cortex lamination, in the fetal age of around midgestation, vascularize the cerebral cortex, radially invading the neural wall from the pial surface to the deeper subcortical layers. Their endothelial lining is characterized by a strong reactivity to fundamental metabolic and efflux barrier-specific transporters, such as the glucose transporter isoform 1, Glut-1, and to a member of the ABC (ATP-binding cassette) superfamily, the multidrug transporter P-glycoprotein (P-gp or ABCB1). On double staining with Glut-1 and the astroglia marker GFAP, radial vessels appear surrounded by a curtain of GFAP-reactive RG fibers and their ECs express high levels of Glut-1 (Figures 2A–C). Cortex stem microvessels appear aligned with rows of neuroblasts and are seen to form their first collaterals, stem vessels and branches being revealed by the endothelial expression of P-gp (Figures 2E,F). The presence of two BBB transporters indirectly implies that tight junction (TJ) formation is in progress in ECs and that the barrier is partially functional. In fact, on double staining with P-gp and the TJ protein occludin, the latter appears highly expressed on both stem vessels and collaterals, and shows a linear, junctional staining pattern (Figures 2D–F). Also on double staining with the TJ protein claudin-5, occludin displays a clearly junctional distribution, whereas a punctate cytoplasmic pattern still prevails for claudin-5 (Figures 2G–I). Overall, these data demonstrate an ongoing process of BBB differentiation in human cerebral cortex microvessels at midgestation.

### Vessel-specific contacts occur between CXCL12-reactive RG fibers and cortex microvessels

**Figure 3:**
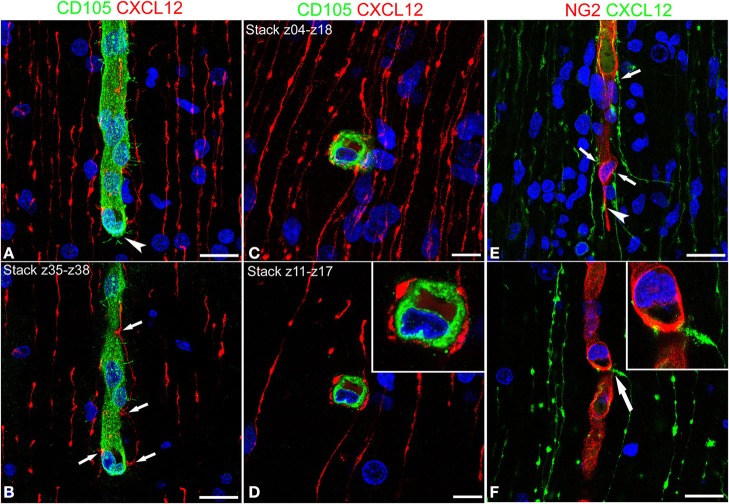
**Confocal images of human cerebral cortex at midgestation double labeled with CD105/CXCL12 (A–D) and NG2/CXCL12 (E,F)**. CXCL12-reactive RG fibers show evenly spaced varicosities **(A)** that make contacts at regular intervals (better recognizable on the selection of optical planes z35–z38; **B**, *arrows*) with a CD105 labeled, perforating cortex microvessel; note in **(A)** the tip endothelial cell on the growing vessel front (*arrowhead*). **(C,D)** Transverse view of a vascular branch completely surrounded by CXCL12 RG fibers, whose intimate relationships are better recognizable on a shorter stack of optical planes (z11–z17, **D**; *inset*). **(E)** Multiple CXCL12-reactive varicosities touching the NG2 pericyte cover (*arrows*) and a pericyte leading process (*arrowhead*); **(F)** One CXCL12-labeled varicosity bents to contact the vessel wall (*arrow* in **F**; *inset*). Bars: **A,B** 20 μm; **C,D** 10 μm; **E,F** 20 μm.

Cerebral cortex microvessels are also revealed by the endothelial marker CD105, which also allows recognition of the growing front of the invading vessels, that is characterized by CD105-reactive endothelial tip cells (Figures 3A,B). On double immunostaining for CXCL12, CD105 labeled microvessels appear surrounded by palisades of CXCL12-reactive RG fibers and in direct contact with sequences of swellings characterizing the RG fibers (Figures 3A,B) that are also seen in close contact with radial vessel collaterals (Figures 3C,D). Double staining with proteoglycan NG2 antibody as a pericyte marker also demonstrates several points of apposition between regular sequences of RG vascular varicosities and the microvessel wall (Figures 3E,F).

**Figure 4:**
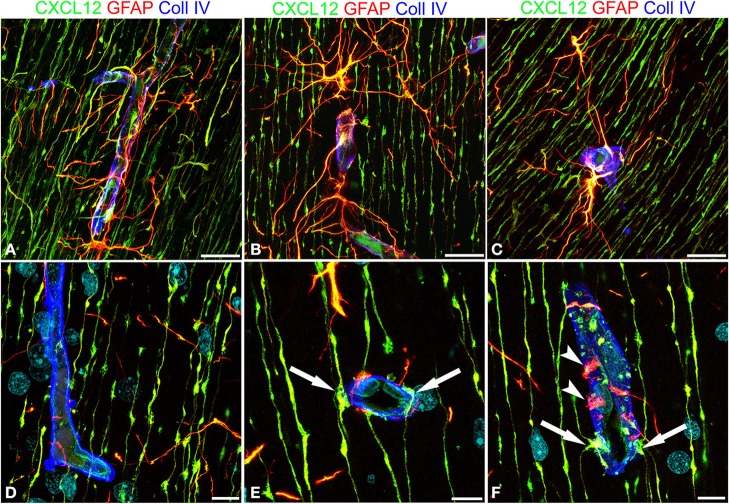
**Confocal images of human cerebral cortex at midgestation after multiple labeling with CXCL12, GFAP, and Coll IV**. **(A–C)** GFAP-CXCL12-reactive perivascular astrocytes and CXCL12-reactive RG cells contributing perivascular endings to the microvessel wall revealed by collagen IV (*blue*). **(D**–**F)** On selected single optical planes the RG vascular contacts are distinguishable, two *en passant, symmetrical* varicosities on the vessel wall (**E**, *arrows*) and two omega-shaped *asymmetrical* varicosities on either sides of the vessel profile (**F**, *arrows*); note in **(F)** classical GFAP-labeled astrocyte end-feet (*arrowheads*). Bars: **A–C** 20 μm; **D–F** 10 μm.

**Figure 5:**
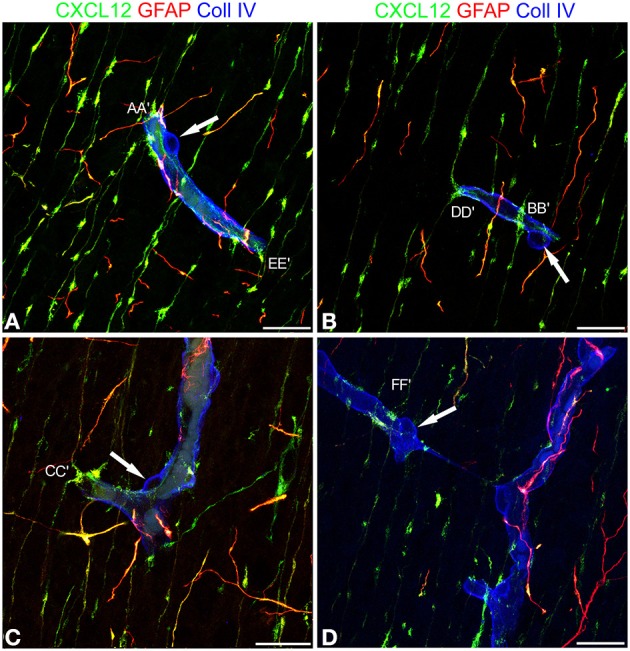
**Confocal images of human cerebral cortex at midgestation after multiple labeling with CXCL12, GFAP, and Coll IV**. **(A–D)** A further collection of CXCL12-reactive RG varicosities in contact with the vessel wall, whose Coll IV staining also shows the bulging nuclei of pericytes (*arrows* in **A**–**D**). Single RG-vascular contacts are recognizable in these pictures **(AA′, BB′, CC′, DD′, EE′, and FF′)** and are shown as enlarged details in Figure 6. Bars: **A–D** 20 μm.

**Figure 6:**
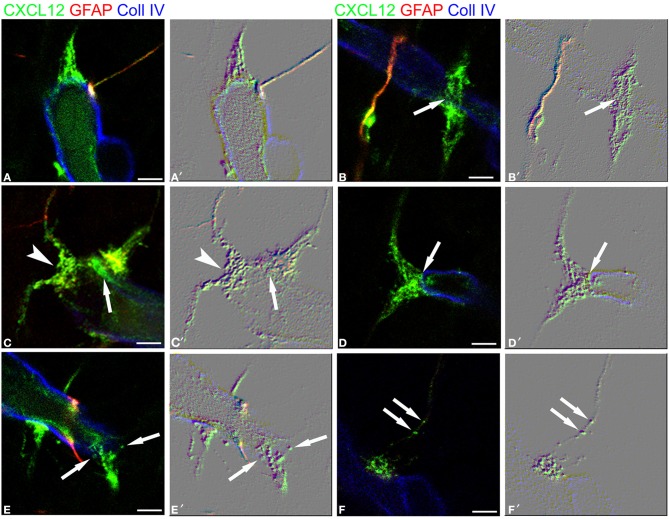
**RG-vascular contacts shown in Figure 5, (A–F) and the same pictures rendered with an embossed image filter (A′–F′)**. The profile in relief of MV-like subcellular structures is amplified and well-recognizable on the flat surface; note in **(A,A′)** gathered MVs, in **(B,B′)** an *en passant, symmetrical* varicosity (*arrow*), in **(C,C′)** an *asymmetrical* varicosity (*arrowhead*) and an endothelial CXCL12-reactive density faced by a RG contact (*arrow*), in **(D,D′)** a clearly recognizable MV (*arrow*), in **(E,E′)** released MVs (*arrows*), and in **(F,F′)** MVs within the RG fiber (*arrows*). Bars: **A–F** 2.5 μm.

These first observations were extended by triple immunostaining with GFAP and CXCL12, as a classical and a novel marker of RG cells, respectively, and with collagen type IV (Coll IV) as a marker of the vascular basal lamina (Figures 4A–F). By this staining, in the subcortical layers, where the earliest astrocytes become recognizable, the microvessels appear surrounded by a perivascular subset of astrocytes that express high levels of both GFAP and CXCL12 (see also Virgintino et al., 2013) (Figures 4A,B). On the same sections, RG fibers reveal a lower GFAP immunostaining and a higher staining of CXCL12 (Figures 4A–C). The two markers colocalize on RG vessel-specific varicosities that make *en passant* contacts maintaining a symmetrical profile, denoted as *symmetrical* varicosities, or assume an omega-like shape and are therefore indicated as *asymmetrical* varicosities (Figures 4D–F). In favorable conditions of tissue and antigenicity preservation, and with the opportune setting of laser potency and confocal parameters, the described RG *symmetrical* and *asymmetrical* varicosities appear filled by CXCL12-labeled dot-like structures that sometimes display a microvesicular nature (Figures 5A–D, 6A–F,A′,F′). The CXCL12 MVs are densely packed within varicosities measuring about 1–1.5 μm and show a diameter of 100–200 nm (Figures 5A–D, 6A–F,A′–F′). At sites of vascular contact, CXCL12 densities are also recognizable beneath the Coll IV basal membrane (Figure 6C).

### RG varicosity and MVs are clearly revealed by transmission electron microscopy

**Figure 7:**
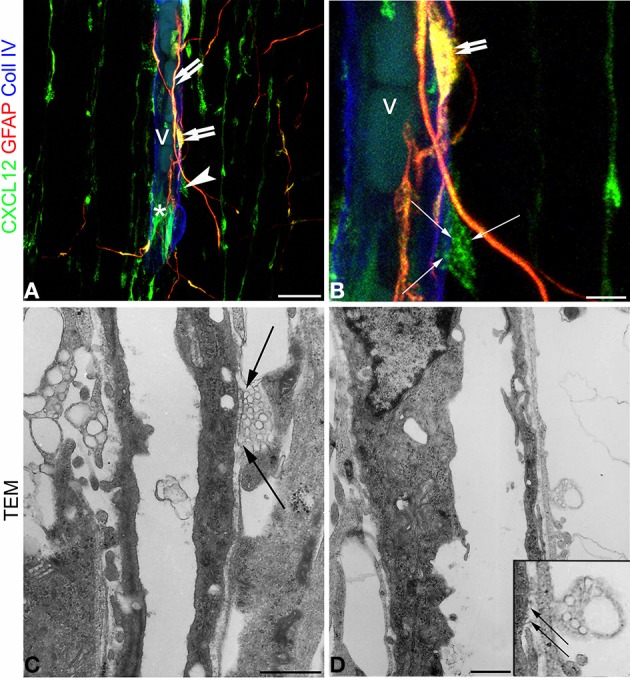
**Confocal images (A,B) and transmission electron microscopy (C,D) of human cerebral cortex at midgestation. (A,B)** Triple labeling with CXCL12, GFAP, and Coll IV shows the profile of a radial vessel (v) contacted by GFAP/CXCL12-reactive perivascular astrocyte processes (*double arrows*) and CXCL12-reactive RG fibers (*asterisk*); a RG varicosity (*arrowhead*) in contact with the vessel wall is enlarged in (**B**) and appears filled by CXCL12 labeled MVs (*arrows*). (**C**,**D**) At ultrastructural level, perivascular RG varicosities show their content of membrane-bound MVs regularly aligned with the vessel contact side (**C** and *inset* in **D**; *arrows*). Bars: **A** 20 μm; **B** 5 μm; **C,D** 5 μm.

When comparing confocal images of radial microvessels, after triple immunostaining for CXCL12, GFAP, and Coll IV (Figures 7A,B), with similar fields studied by transmission electron microscopy (TEM), some details, appreciated in the former, become clearly defined by ultrastructural observations (Figures 7C,D). The vesicular nature of the subcellular structures observed by confocal microscopy after CXCL12 immunolabeling is confirmed (Figures 7A–D), while RG varicosities and the vascular basal membrane are seen in close contact at several points of the vessel wall (Figures 7C,D). MVs appear homogeneous in size and aligned against the varicosity membrane facing the vessel contact site (Figures 7C,D).

### Gap junction protein Cx43 colocalizes with chemokine CXCL12 in RG varicosities

**Figure 8:**
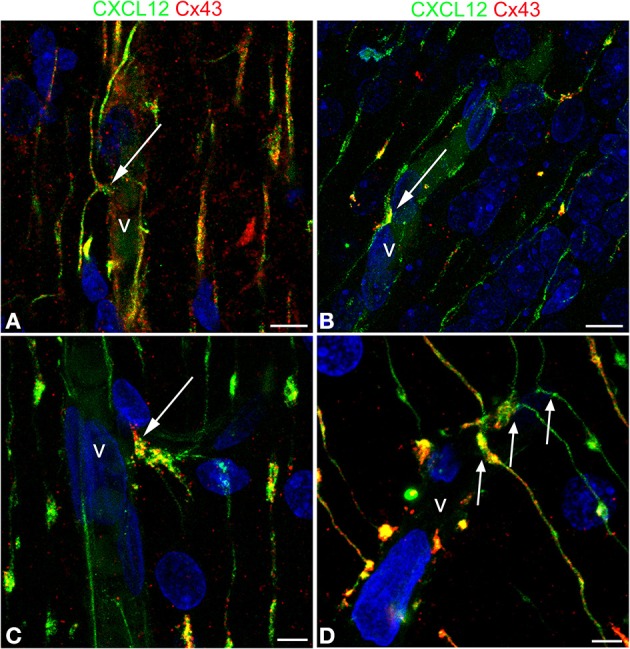
**Confocal images of human cerebral cortex at midgestation double labeled with CXCL12 and Cx43**. **(A–D)** Examples of *omega-shaped*/*asymmetrical* varicosities (*arrows*) in contact with the vessel wall (v) characterized by CXCL12/Cx43 colocalization; note in **(D)** the regular sequence of deflected RG fibers forming asymmetrical contacts (*arrows*). Bars: **A,B** 10 μm; **C,D** 5 μm.

The presence of Cx43 was revealed in double immunostaining for CXCL12 and Cx43, the rationale for this approach being sustained by data on Cx43 involvement in RG-guided neuronal migration (Elias et al., 2007; Matsuuchi and Naus, 2013) and by studies on Cx43 in RG cells (Virgintino et al., 2001; Errede et al., 2002). Cx43 plaques are revealed along the RG fibers and concentrate in vessel-contacting RG varicosities, where Cx43 extensively colocalizes with CXCL12 (Figures 8A–D).

## Discussion

### Radial glial cell varicosities and CXCL12 MVs involved in cerebral cortex vascularization

In 1887, when Giuseppe Magini utilized for the first time the Golgi impregnation technique on mammalian developing cerebral cortex, he described “neuroglial cells with very long and thin filaments bearing numerous varicosities or swellings” (Bentivoglio and Mazzarello, 1999). Subsequently, in 1904, Cajal confirmed the existence of radiating fibers and extended Magini's work, definitely describing “swelling intercalated along the radial glia fibers protoplasmic accumulation” (Ramón y Cajal, 1904). In recent studies on neurogenic RG in rat embryos, DiI-labeled RG cells have shown a “Golgi-like” morphology, including the small varicosities, that have been described as points of cytoplasm accumulation in the flux and reflux of fluid through the cell during mitosis (Weissman et al., 2003; Kriegstein and Alvarez-Buylla, 2009). Surprisingly, CXCL12, a chemokine that accumulates in RG fibers, can show these cellular structures in detail, going beyond the mere profile of cytoplasmic swelling, and instead suggesting a role as specialized cellular sites. RG cells, like cerebellar Bergmann glia and retinal Müller cells, both examples of RG-like glial cells in adult CNS, may display a differential distribution of organelles and membrane domains along their length (Fedoroff and Vernadakis, 1986). The described RG varicosities can be regarded as special compartments of the cell devoted to closely adjoining the vessel wall to form specific vascular contacts.

CXCL12-bearing MVs, as cell-derived MVs (diameter ranging from 0.1 to 1 μm) involved in cell-to-cell communication, may directly bud from the plasma membrane to convey their membrane-associated bioactive molecule to the target cells (György et al., 2011; Frühbeis et al., 2012; Kalra et al., 2012). It has been demonstrated that cultured astrocytes shed MVs from cell domains interpreted as astrocyte end-feet, which contain angiogenic factors, FGF-2, and VEGF, and membrane associated ß1-integrin (Proia et al., 2008), MVs can, therefore, participate in angiogenic events, transferring pro-angiogenic factors to ECs (Boulanger and Tedgui, 2005; Muralidharan-Chari et al., 2010; Martinez and Andriantsitohaina, 2011). Moreover, it has also been shown that tumor-derived MVs released by tumor ECs can be taken up by normal ECs through endocytosis, and promote motility and tube formation, conferring proangiogenic properties to quiescent ECs (Kawamoto et al., 2012). It has been demonstrated that in zebrafish brain and in rat developing retina, chemokine CXCL12 signaling specifically controls the correct pathfinding of newly formed microvessels (Strasser et al., 2010; Unoki et al., 2010; Bussmann et al., 2011; Fujita et al., 2011). Our findings support these roles, suggesting that the chemokine can become available, through the shedding of MVs from RG varicosities, for ligand/receptor interactions on ECs or, alternatively, after MVs endocytosis, for direct EC activation.

### CXCL12 vessel-specific activity regulated by Cx43 non-channel roles

The preliminary results that show a CXCL12/Cx43 colocalization in RG glio-vascular contacts suggest that the release of CXCL12 can be regulated by the activation of Cx43 hemichannels, non-junctional structures that can be involved in the release of signaling molecules, and that are a common attribute of cells during development, forming functional hexameric rings before proper gap junctions appear (Nagy et al., 2004; Nielsen et al., 2012; Stehberg et al., 2012; Giaume et al., 2013; Zhou and Jiang, 2014). In fact, during cerebral cortex development, Cx43 displays channel-independent roles also when regulating neuroblast migration (Matsuuchi and Naus, 2013). Moreover, as demonstrated for human bone marrow stromal cells, the secretion of functional CXCL12 by RG cells could be mediated by Cx43 (Schajnovitz et al., 2011), and Cx43 has been found to regulate CXCL12-mediated Rap1 EC spreading “*in vitro*” (Machtaler et al., 2011).

### Activation and cooperation of multiple pro-angio-morphogenetic pathways

The emergence of the chemokine system as a key signal during CNS development and vascularization raises the issue of a possible functional interaction with other pro-angiogenic pathways such as the canonical Wnt/β-catenin pathway, whose involvement in regulating vascular morphogenesis has already been described, together with a CNS-specific angiogenic, and barriergenesis function in concert with other signaling pathways (Daneman et al., 2009; Dejana, 2010; Reis and Liebner, 2013). As suggested by studies on the effect of RG cells ablation, a cooperative effect of CXCL12/CXCR4–Wnt/β-catenin exists during brain vascularization. In fact, in absence of RG cells, a significant reduction has been observed of cortical thickness, and the regression of nascent brain vessels, *via* the inhibition of EC Wnt signaling in a contact and stage-dependent manner (Ma et al., 2013). Moreover, the interaction between Wnt/β-catenin signaling and the chemokine receptors CXCR4 and CXCR7 leads to a coordinated cell migration *via* differential receptor regulation (Aman and Piotrowski, 2008); this same cooperation may generate localized molecular cues, inducing differentiation of BBB properties and cortex microvessel maturation (Engelhardt and Liebner, 2014).

In conclusion, this study, carried out during human cerebral cortex development, supports the concept of an involvement of RG cells in the synchronized regulation of neuro-vascular patterning and early BBB differentiation. According to our model (Figures 1A–C), this may take place thanks to the unique, spatial distribution of RG cells, the varicosity/MV/CXCL12/Cx43 machinery, and the help of an overlapping repertoire of signaling molecules (Carmeliet and Tessier-Lavigne, 2005; Vasudevan and Bhide, 2008; Tam and Watts, 2010).

## Author contributions

Mariella Errede Substantial contribution to the design of the work, acquisition, electron microscopy analysis, interpretation of data. Francesco Girolamo Substantial contribution to the design of the work; acquisition, confocal microscopy analysis, interpretation of data. Marco Rizzi Substantial contribution to acquisition and confocal microscopy analysis, interpretation of data. Mirella Bertossi Substantial contribution to interpretation of electron microscopy data and revising the work critically for important intellectual content. Luisa Roncali Substantial contribution in revising the work critically for important intellectual content and final approval of the version to be published. Daniela Virgintino Substantial contributions to the conception of the work, drafting the work and revising it critically; final approval of the version to be published.

### Conflict of interest statement

The authors declare that the research was conducted in the absence of any commercial or financial relationships that could be construed as a potential conflict of interest.

## Abbreviations

BBB: blood-brain barrier
CD105: endoglin
CNS: Central Nervous System
Coll IV: collagen type IV
Cx43: connexin 43
CXCL12: C-X-C motif chemokine 12
EC: endothelial cells
GFAP: glial fibrillary acidic protein
Glut-1: glucose transporter isoform 1
MVs: microvesicles
NG2: nerve/glial antigen 2
P-gp: P-glycoprotein
RG: radial glia
TJ: tight junction.
